# Using Free Navigation Reference Points and Prefabricated Bone Plates for Zygoma Fracture Model Surgeries

**DOI:** 10.1007/s40846-016-0144-x

**Published:** 2016-06-06

**Authors:** Tien-Hsiang Wang, Hsu Ma, Ching-Shiow Tseng, Yi-Hong Chou, Kun-Lin Cai

**Affiliations:** Division of Plastic and Reconstructive Surgery, Department of Surgery, Taipei Veterans General Hospital, Taipei, 11217 Taiwan; Department of Mechanical Engineering, National Central University, 300 Jhongda Road, Jhongli District, Taoyuan, 32001 Taiwan; Department of Radiology, Taipei Veterans General Hospital, Taipei, 11217 Taiwan; School of Medicine, National Yang-Ming University, Taipei, 11221 Taiwan

**Keywords:** Navigation, Computer-assisted surgery, Fracture fixation, Surgical planning, Image processing, Virtual reality, Registration

## Abstract

Surgical navigation systems have been an important tool in maxillofacial surgery, helping surgeons create a presurgical plan, locate lesions, and provide guidance. For secondary facial bone reductions, a good presurgical plan and proper execution are the key to success. Previous studies used predetermined markers and screw holes as navigation references; however, unexpected situations may occur, making the predetermined surgical plan unreliable. Instead of determining positions preoperatively, this study proposes a method that surgeons can use intraoperatively to choose surface markers in a more flexible manner. Eight zygomatic fractures were created in four skull models, and preoperative computed tomography (CT) image data were imported into a self-developed navigation program for presurgical planning. This program also calculates the ideal positions of navigation references points for screw holes. During reduction surgery, markers on fractured bone are selected, registered, and calculated as free navigation reference points (FNRPs). The surface markers and FNRPs are used to monitor the position of the dislocated bone. Titanium bone plates were prefabricated on stereolithography models for osteosynthesis. Two reductions with only FNRPs, as well as six reductions with FNRPs and prefabricated bone plates, were successfully performed. Postoperative CT data were obtained, and surgical errors in the six-reduction group were evaluated. The average deviation from the screw hole drilling positions was 0.92 ± 0.38 mm. The average deviation included displacement and rotation of the zygomas. The mean displacement was 0.83 ± 0.38 mm, and the average rotations around the x, y, and z axes were 0.66 ± 0.59°, 0.77 ± 0.54°, and 0.79 ± 0.42°, respectively. The results show that combining presurgical planning and the developed navigation program to generate FNRPs for assisting in secondary zygoma reduction is an accurate and practical method. Further study is necessary to prove its clinical value.

## Introduction

In most secondary facial bone fractures, it is challenging to restore the anatomic positions of dislocated bone. The fracture lines fuse together and leave few clues for reduction. In panfacial and bilateral fractures, the problem is more complicated as there is no normal side for reference. Therefore, a good presurgical plan is important for selecting the correct pathways for osteotomy and manipulation.

Surgical navigation systems and computer-aided design and computer-aided manufacturing (CAD/CAM) have been applied in neurosurgery, orthopedics, and maxillofacial fields [1–6]. Several kinds of navigation system have been developed for clinical application, including mechanical, electromagnetic, and optical systems [4–9]. The most widely used system for maxillofacial surgery is the optical navigation system, which helps to increase precision. For secondary facial bone reductions, combining surgical navigation with high-resolution computed tomography (CT) imaging and CAD/CAM is helpful. These technologies are beneficial for preoperative planning, guiding surgical instruments, and evaluating surgical results [8–11]. These procedures can be summarized as follows: (1) acquiring preoperative image data from patients and performing preoperative simulation; (2) using the planning results to monitor and assist in reduction surgery; and (3) evaluating intraoperative or postoperative results. Among these, navigational assistance plays an important role.

To transfer the spatial information from the object to the navigation system, ideally, dynamic reference frames (DRFs) should be applied over the skull and deviated bone. These DRFs continue to provide the object coordinates to the navigation system, and the real-time bone positions can be synchronized with those in virtual reality. Just like using the Global Positioning System for vehicles, surgeons can ‘drive’ the fractured bone into its planned position. However, the DRF is almost always too large for the reduction procedure. Even if the setup were possible, the DRF would be obstructive and cause inconvenience. Researchers have developed several solutions. Klug et al. [8] used stereolithography (SLA) models to perform presurgical planning for zygoma osteotomies and reductions; the result was pre-bent bone plates and planned screw positions. During the real operation, they used a navigation system to locate the predetermined screw holes and the pre-bent bone plates for osteosynthesis. Similar procedures include obtaining preoperative CT scan data from patients for simulation surgery with/without SLA model surgery, and using the results for the real operation. In these situations, the predetermined surface marks or screw holes on the bone serve as navigation reference points. Operators can use a DRF-mounted probe to check whether the mark positions are in good alignment with the presurgical plan [11, 12].

In these solutions, surgical plans are created before surgery, but the situation may change during surgery. The fractured bone may be so small that no optimal place to drill can be found or the soft tissue dissection may not expose enough space for drilling. The vibration and drilling force of the instrument can sometimes break fragile bone, especially if there is a subtle fracture. To handle these unexpected situations, this study develops a method for selecting navigation reference points during surgery, instead of using predetermined surface marks. These points are called free navigation reference points (FNRPs). Similar to the above-mentioned methods, the FNRPs can be used during real-time surgery for navigation reference. Furthermore, pre-bent bone plates are used for assisting osteosynthesis. This study uses artificial skull models with eight zygoma fractures to evaluate the effects of FNRPs created using a self-developed navigation program in two situations: one with only FNRPs and one with FNRPs and pre-bent bone plates. The results demonstrate that the proposed method is reliable and useful.

## Materials and Methods

### Preparation of STL Images

Four artificial skull models from two companies (Synbones Skull #8411, SYNBONE AG, Malans, Switzerland; Sawbones Full Skull #1345-28/#1345-20, Pacific Research Laboratories Inc., Vashon, WA), two zygoma fractures in each skull model, were used for the surgery model, and the fracture line edges were deliberately widened to eliminate possible clues of bone reduction. The fractured zygomas were fixed with a hot melted adhesive. 0.5-mm-thick slices (Aquilion 64, Toshiba Medical Systems Corporation, Otawara, Japan) were created, and then a skull CT scan was obtained in the Department of Radiology at Taipei Veterans General Hospital. The two-dimensional CT data were reconstructed into three-dimensional (3D) stereolithography (STL) images using image-processing software (Amira version 4.0, TGS, Berlin, Germany). The purpose of this study was to test the accuracy of the proposed method, so the non-dislocated side was not used as a target for reduction. The engineer and surgeon used a CAD program (Geomagic Studio 8, Raindrop Geomagic, Inc, Research Triangle Park, NC) to free the fractured portions and then reduced the dislocated zygomas into ideal positions.

### Development of Navigation Program for Presurgical Planning

First, the preparation work was done in virtual reality. Moving the zygoma from its dislocated site to the reduced site by matching the point clouds on the zygoma surfaces with an iterative closest point (ICP) algorithm method [13], the spatial information was automatically recorded by the self-developed navigation program. This program was developed by our engineers using Visual C^++^ (Microsoft Visual C^++^ version 10.0.40219, Microsoft Corporation, Redmond, WA) with a Visualization Toolkit (VTK) software system [14]. The recorded information was a 4 × 4 matrix that included the rotational angle and translational distance. Surgeons can use this program for surface mark registration, transformation matrix generation, and surgical simulation. Using the matrix calculation in this program, the coordinates of every point on the dislocated zygoma could be transformed into its corresponding coordinates on the reduced zygoma:

1 $${\text{P}}_{\text{R}} = {\text{ T}}_{\text{D}}^{\text{R}} {\text{P}}_{\text{D}}$$

where T_D_^R^ is the transformation matrix, P_D_ is the position on the dislocated zygoma, and P_R_ is the new position that will be on the reduced zygoma. The coordinates of any selected point on the dislocated zygoma can be input into the program, and its new reduced coordinates on the reduced zygoma will be displayed on the monitor.

Accordingly, the inverse matrix of T_D_^R^, (T_D_^R^)^−1^, can transform the coordinates of a reduced point into its previously dislocated position. For example, in presurgical planning, we could reduce and fix the dislocated zygoma in virtual reality and then back-calculate the sites for the bone plate screw holes for real fixation:

2 $${\text{P}}_{\text{D}} = \, \left( {{\text{T}}_{\text{D}}^{\text{R}} } \right)^{ - 1} {\text{P}}_{\text{R}}$$

**Fig. 1:**
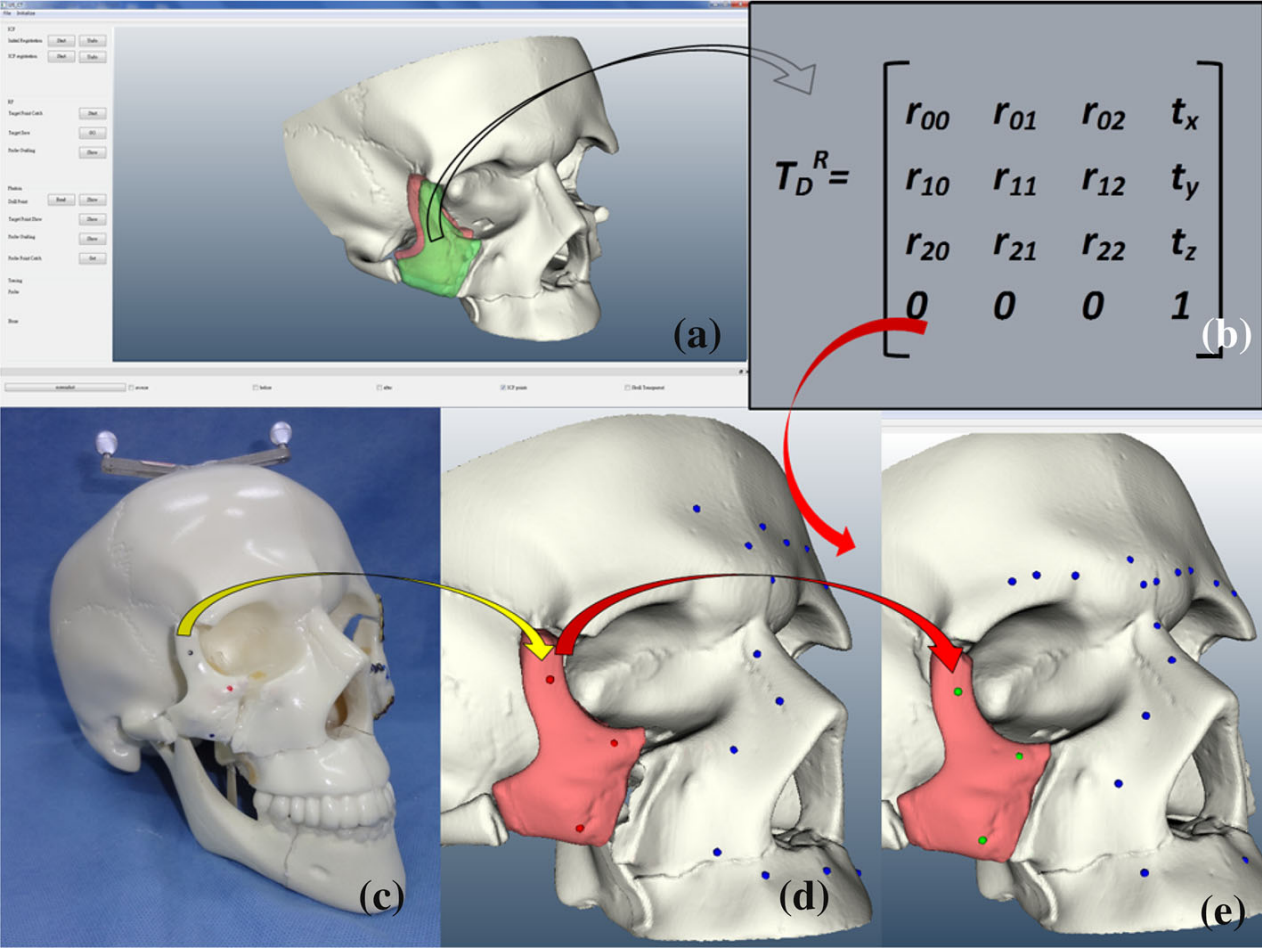
Generation of FNRPs. **a** STL image of skull model was imported into navigation program, and dislocated zygoma (*red portion*) was reduced into its ideal position (*green portion*). **b** Transformation matrix was generated automatically from our program, which recorded the virtual displacement and rotation data from manipulating zygoma (*hollow arrow*). **c** We selected three points on dislocated zygoma and registered them. Yellow arrow indicates navigation data transmission. **d** and **e** In virtual reality, every point coordinate on dislocated zygoma (*red dots* in **d**) can be transformed into its reduced position (*green dots* in **e**) through matrix transformation (*red arrows*). *Green dots* are FNRPs

If we were to drill a hole in a dislocated zygoma and perform the transformation matrix calculation, we could find the reduced position for that hole. Then, the reduced position would be an FNRP that could be used as a navigation target during reduction surgery (Fig. 1). This is because if we monitored the hole position using the navigation system, we would know how the discrepancy by judging the distance between the hole and the FNRP. To confirm the position of an object in a 3D environment, at least three FNRPs are necessary. Selecting surface marks and performing the registration should be done before fracture reduction, as surgical manipulation would break the zygoma-skull relationship, and any subsequent registration would no longer be reliable.

### Situation One: Reductions with only FNRPs

**Fig. 2:**
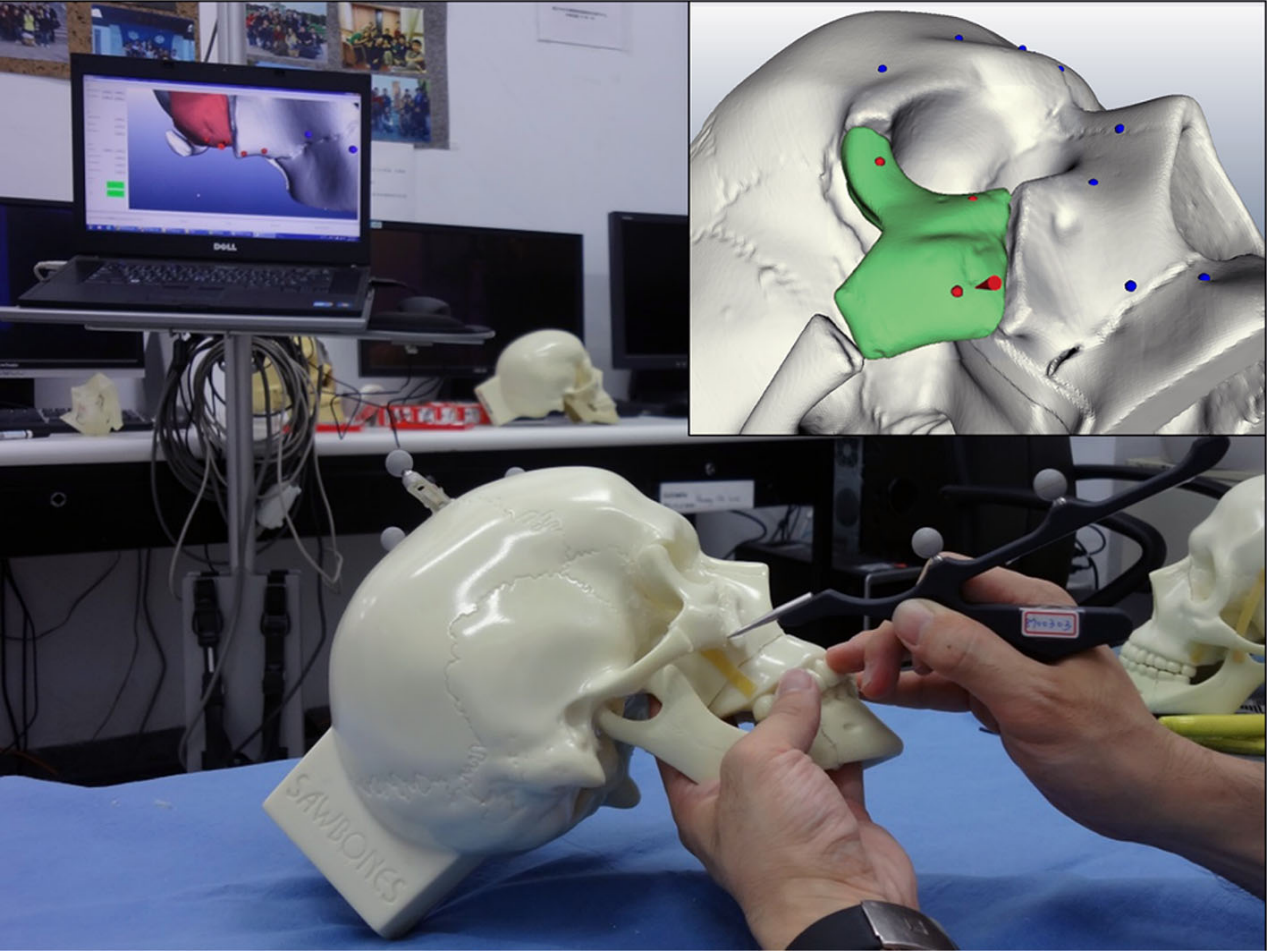
Probing procedure. Surgeon used navigation probe to tap already registered point, and relation between the point and its FNRP was shown on screen. *Inset* shows cone object indicating relative position of registered point in virtual reality; *red point* near cone object is FNRP of that point

For skull model surgery, the first situation used FNRPs for assisting in the zygoma reduction. A DRF was fixed to the parietal skull, and then surgeons performed the registration. The initial coarse alignment with the optical navigation system (Polaris Spectra, Northern Digital Inc., Waterloo, Canada) was accomplished by using three points over the nasion and the bilateral zygomatico-frontal suture. Fine alignment was done by choosing 20 random points over the frontal skull, maxilla, and naso-orbital regions. The surgeons chose and marked three points on the surface of the displaced zygoma, located near the zygomatico-frontal suture, the mid-inferior orbital rim, and the lower zygomatic buttress, respectively. This must be conducted before formal reduction. These three points were registered, and after the transformation matrix calculation, their reduced positions were presented on the navigation system screen. These three reduced positions were used as FNRPs. After registration, the fractured zygoma was freed and the reduction procedure was begun. We used a navigation probe to monitor the mark positions on the dislocated zygoma; the distance between a marked point and its relative FNRP was used to judge accuracy. This procedure is called a ‘probing’ procedure (Fig. 2). For zygoma fixation, we first applied and fixed four-hole titanium bone plates (MINI 2000, MONDEAL Medical Systems GmbH, Mühlheim, Germany) over the non-dislocated side of the zygomatico-frontal suture, mid-inferior orbital rim, and lower zygomatic buttress; then, we bent and adjusted the plates and fixed them over the dislocated side after confirming the zygoma position using the probing procedure. We performed the initial two-zygoma reductions on a bilaterally fractured skull model. After the operation, we checked the skull CT scan again and compared the STL images for the presurgical plan and reduced skulls. The non-fractured skull portions were matched using an ICP algorithm method, and the discrepancy between the two zygomas was visually evaluated. The experience gathered from this technique was applied for the main part of the surgery.

### Situation Two: Reductions with Prefabricated Bone Plates and FNRPs

The second situation combined the use of prefabricated titanium plates and FNRPs for the zygoma reductions. From the first part of the surgery, we knew that although we could monitor the intraoperative positions of a fractured zygoma by surface markers and FNRPs, keeping a stable support during reduction and plating would be difficult and time-consuming. Therefore, we used pre-bent bone plates to assist in reduction. The procedures for preparing the zygoma fractures in the skull model, preparing the STL images from the CT scan data, and creating a presurgical plan were the same as those in the preliminary surgery. A real SLA model that was produced using a rapid prototyping (3D printing) method (SCS 8100, Sony Manufacturing Systems Ltd, Tokyo, Japan) with zygomas in reduced positions was used for bone plate prefabrication. A DRF was fixed on the cephalic portion of the SLA model, and the operator selected screw holes for bone plate fixation. The plates were bent according to the curvature across the fracture sites over the zygomatico-frontal suture, mid-inferior orbital rim, and lower zygomatic buttress. There were three bone plates and 12 screws for each zygoma. Then, the plates and screws were removed, and every screw hole was registered. To decrease deviation errors, care was taken to keep the probe tip just on the superficial level of the holes. The screw hole positions were back-calculated by our program [using the inverse matrix in Eq. (2)] to generate the points for the fractured sites.

**Fig. 3:**
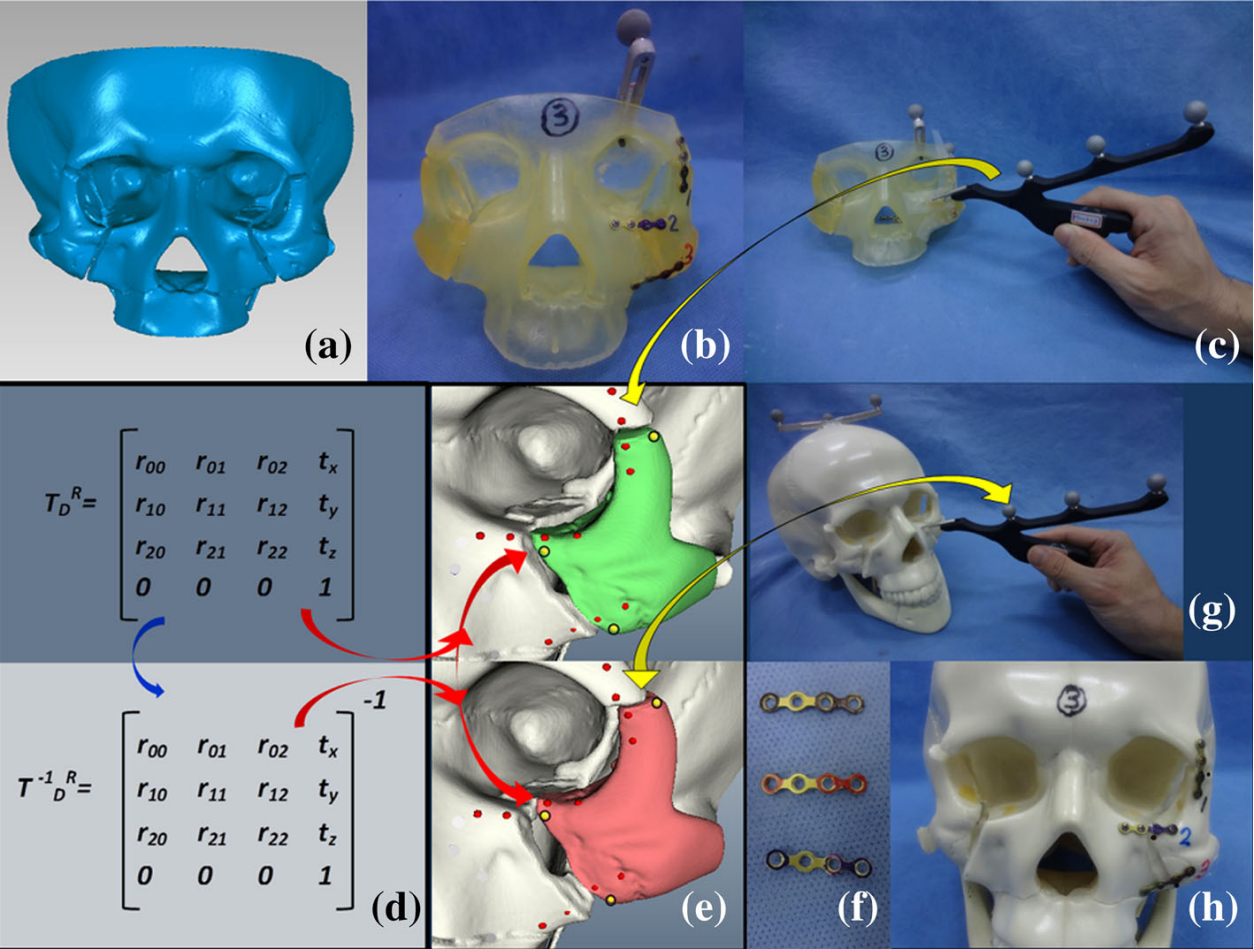
Use of FNRPs and pre-bent bone plates for zygoma reduction model surgery. **a** STL image with bilateral zygoma fractures in reduced position. **b** SLA model produced from STL image. Bone plates were already fixed on it. *Screw holes* were determined by surgeon. **c** Registration of *screw holes* after removal of bone plates and screws. *Yellow arrows* indicate navigation data transmission. **d** Transformation matrix (*top*) and inverse of transformation matrix (*bottom*). **e** Inverse matrix can be used to back-calculate *screw hole* positions on dislocated zygoma (*bottom*, *red* *dots on red zygoma*) from positions that had just been registered (*top, red dots on green zygoma*). *Red arrows* indicate coordinate data transformation. **f** Pre-bent bone plates from SLA model. **g** Navigation system was used to mark and create *screw holes*. Surgeon selected three marks on zygoma and registered them (**e**, *yellow dots on red zygoma*), and FNRP positions were calculated (**e**, *yellow dots on green zygoma*). **h** Zygoma was reduced with guidance of FNRPs and bone plates

During the skull model surgery, a DRF was set on the parietal skull. With the help of the navigation system, screw holes were drilled according to the positions that were previously determined. The screw holes were registered soon after drilling. As in situation one, the operator selected three surface markers on the zygoma and the navigation program calculated their FNRPs. Then, the surgeon freed the zygoma and monitored the reduction using a probing procedure, and the prefabricated bone plates were applied and fixed. A total of six zygoma fracture reductions were performed for the skull models. The procedures are summarized in Fig. 3.

### Evaluation of Errors in Situation Two

The errors from the drilling procedures were dependent on the skill and experience of the operators and were easy to measure. After we drilled a screw hole according to the presurgical plan, we soon registered that hole. The distance between the registered screw hole position and its predetermined position in the presurgical plan was defined as the drilling error.

Errors could also be derived from the whole procedure. The reduced skulls were sent for CT scans, and the data were reconstructed into 3D STL images. At this moment, the non-operating parts of the preoperative and postoperative STL images were matched using an ICP algorithm; there were translation and rotation differences between the preoperative planned zygoma and reduced zygoma. The translational error was defined as the distance between the two geometric centers of the two zygomas, and the rotational error was obtained by calculating the degrees of rotation among the xyz coordinate axes of the two zygomas.

## Results

**Fig. 4:**
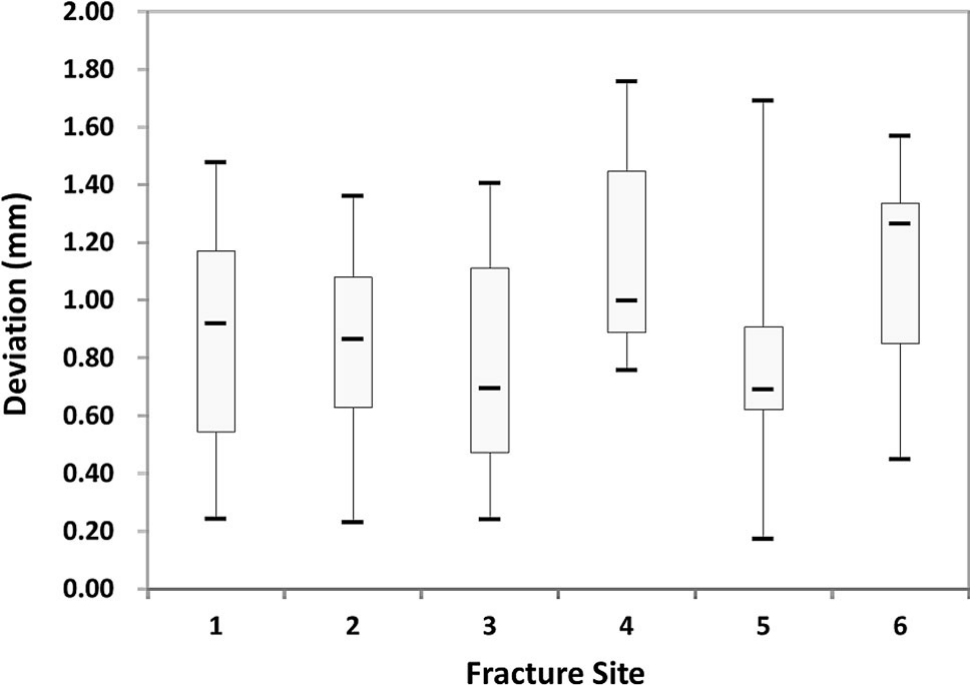
Distribution of deviation errors for drilling procedure. All deviations were < 2 mm, and most were between 0.4 and 1.5 mm

From January 2013 to October 2013, we performed eight zygoma reductions in four skulls using the proposed method. The two results in situation one were visually evaluated by comparing the STL images of the surgical plan and the postoperative skull; the result was satisfactory. Six reductions were performed using FNRP assistance and pre-bent bone plates. We evaluated deviations from the drilling procedures and global procedures for these six reductions. The errors from the drilling procedures are listed in Table 1. For the six zygoma fractures and 72 total screw holes, the average displacement was 0.92 ± 0.38 mm (Fig. 4). The errors from all procedures include differences in distances and rotational angles between the planned zygomas and reduced zygomas (Table 2). The average displacement was 0.83 ± 0.38 mm; the mean absolute rotational values around the x, y, and z axes were 0.66 ± 0.59°, 0.77 ± 0.54°, and 0.79 ± 0.42°, respectively.

**Table 1:** Deviation of drilling procedure

Fracture site	1	2	3	4	5	6
Deviation	0.977	0.231	0.760	0.759	1.025	0.911
	0.748	0.914	0.548	0.867	0.871	0.449
	1.032	0.907	1.405	1.017	0.57	1.317
	0.553	1.360	1.066	1.628	1.691	1.294
	0.497	0.655	1.340	0.941	0.666	1.349
	0.522	0.668	0.240	1.241	0.669	0.781
	0.862	1.152	0.633	1.426	1.010	1.235
	1.164	0.822	0.337	0.978	0.626	0.713
	1.188	0.547	1.243	1.757	0.713	1.547
	0.243	0.516	0.767	1.511	0.806	1.568
	1.477	1.054	0.372	0.833	0.174	0.873
	1.295	1.253	0.506	0.895	0.608	1.331
Mean	0.880	0.840	0.768	1.154	0.786	1.114
Total mean ± standard deviation	0.924 ± 0.380					

Deviation (mm) was defined as the distance between the registered screw hole and its planned position

**Table 2:** Evaluation of postoperative error

Fracture site	Displacement (mm)	Rotation (degrees)		
		X axis	Y axis	Z axis
1	0.905	0.505	0.415	−0.139
2	0.375	−0.679	1.433	−0.869
3	1.117	0.038	0.969	1.133
4	0.263	1.858	0.243	1.356
5	1.137	0.124	0.139	−0.342
6	1.225	−0.807	−1.470	−0.951
Absolute value mean	0.837	0.668	0.778	0.798
Standard deviation	0.380	0.599	0.548	0.427

The deviation (mm) was evaluated by matching the STL images with the postoperative skull and preoperative plan. Displacement was defined as the distance between the two geometric zygoma centers. Rotation was the rotational degree of the coordinate axes of the two zygomas

## Discussion

There are three main types of navigation system for clinical, namely mechanical, electromagnetic, and optical systems. These systems have comparable precision and different benefits and drawbacks [15, 16]. A mechanical system uses a mechanical arm that connects to the patient. It is heavy and unwieldy to handle. The signaling device for an electromagnetic system is small and easy to implant, but metallic instruments may interfere with signal transmission. An optical system has good accuracy and is widely used in maxillofacial surgery, but signaling is blocked if there is interference with the light transmission route.

The principal goal of the secondary zygoma reduction surgery is to restore the patient’s bilateral malar height and symmetry. In these cases, fracture lines are fused, which can increase the difficulty of the reduction. With the introduction of CAD/CAM methods, surgeons can use CAD programs and patients’ preoperative CT scans for presurgical planning. Ideal alignment can be established by putting the dislocated bones into their anatomical positions [12]. Using a CAM method can help with prefabricating bone plates for osteosynthesis, manufacturing surgical guide templates for osteotomies, and producing 3D models for simulation surgery [8, 17, 18].

During surgery, it is important to properly transfer the planned surgical information to guide and monitor the reduction procedures. Surgeons have to know exactly what they are doing and where they are going. Dynamic reference frames over the skull and instruments can continually provide real-time information to the navigation system to help maintain good surgical orientation. For the fractured portion, the most reliable method is to set up another DRF, but the DRF itself may cause inconvenience. Direct checking of the malar bone height with a navigation probe is convenient, but the exact bone position is not known [6, 10]. A more precise method is to incorporate presurgical planning and surgical navigation. Surgeons can perform presurgical osteotomies and reductions in virtual reality, prepare prefabricated surgical templates (bone plates), and use a navigation system to locate screw holes for osteosynthesis [8, 12]. However, templates may not be absolutely reliable. With a navigation system, one can precisely locate a screw hole on the surface, but not as easily as the screw-axis direction. During screw tightening, screw holes with wrong axes may drag bone fragments away from their proper position. This may be improved by designing and using a drill guide to create drill holes in a fixed direction. Using surface marks on fractured bone has been suggested by some authors [11, 19]. With navigational assistance, reduction is performed by continuously monitoring the marker positions. This method is inconvenient in that holding the fractured bone without assistance is a problem [12]. The small surgical field and small-sized bone cannot provide sufficient space for maintaining a steady position. In addition, fixation of one fracture site without interfering with any others is difficult.

Furthermore, the drawback of using predetermined surface marks is inflexibility. Once the planned sites for the surface marks have been determined, it was impossible to make further changes in real surgery. During the drilling procedure, fragile bone or an unnoticed fracture may be prone to damage. In these situations, pre-surgically determined marks are no longer useful. Another situation is that the presurgical plan might be compromised because of overlying soft tissue. Surgeons may find it difficult to create or trace surface marks.

Considering that either the surgical templates or only the surface markers are insufficient for surgical precision, combining these two methods should provide convenience and quality. The proposed program uses a transformation matrix that automatically calculates the ideal coordinates of any point on the dislocated bone, providing surgeons with more choices in selecting reference points. Because of immediate matrix transformation, the corresponding target points are generated immediately for navigation. Therefore, we could freely choose any point on the deviated bone and convert it to an FNRP. This allows surgeons more flexibility and comfort to deal with unexpected situations. Xia et al. [12] suggested the use of a matrix to record translational and rotational information for placing the planned bony segment back in its original position, or vice versa. However, the matrix was not used to calculate the position of a selected point. To increase convenience and save time, pre-bent bone plates were used for reduction guidance. The positions of these bone plate screw holes were planned prior to surgery, and then were created on the skull model. With the help of FNRPs and prefabricated bone plates, one can perform zygoma reduction easily and precisely. Alternatively, we can also register screw hole positions and calculate their FNRPs. However, using screw holes may be inconvenient, as they will be covered by bone plates. The screw hole depth can also cause some trouble, because one cannot see a probe tip in a deep hole. Surgeons have to maintain a constant level during the probing procedure. Unexpected situations such as the presence of an unnoticeable, subtle fracture may also occur; in such cases, surgeons can abandon the prefabricated bone plate and use the procedures in situation one. The solution is similar: create stable marks, register and calculate these marks for the FNRPs, and then complete the reduction.

Another issue to be addressed is the surgical errors in situation two. In the proposed method, drill holes and FNRPs are selected by using the surgical navigation system. If we can reduce errors related to the system, the surgical results will improve. The location of the DRF should be close to the fracture site to reduce optical signal errors from the navigation system, fixation of the DRF should be sufficiently secured, and both of the tips of the navigation probe and the FNRPs should be fine enough to improve accuracy. We analyzed two error types in this study; the first resulted from the screw hole creation, and the second resulted from the entire procedure. If we marked and drilled a screw hole, deviation occurred secondary to hand tremor, hand piece vibration, and drill bit rotation. We could calculate the deviation by registering the deviated screw hole and measuring the distance between the corresponding and planned positions. This deviation was the error from the drilling procedure. Analyzing this error type may help surgeons evaluate their technical skill. In this study, the average displacement from drilling procedures was 0.924 ± 0.38 mm. The drilling procedure created some errors, but they were not significant.

The second error type was difficult to measure and may result from different situations, such as the preparation of an STL image, production of an SLA model, prefabricated bone plates, probing procedure, or reduction procedure. The sum of such errors can be measured by evaluating the position of the reduced zygoma that includes object translation and rotation. Previous works evaluated the errors by measuring the discrepancy of screw positions on the fracture site [8, 11]. Considering the fact that the deviation secondary to the rotation of one object may be exaggerated if they are large in size, we measured the deviation distance of the geometric centers and the rotational degree of the xyz-coordinate axes between the preoperative and postoperative zygomas. In the six reduction surgeries, the mean deviation of the geometric centers was no more than 1 mm (0.84 ± 0.38 mm), and all the mean rotational differences of the xyz axes were within 1°. This is comparable to results reported in previous works [8, 11].

This investigation was performed on skull models, which differs from real clinical procedures in several ways. The main differences come from the overlying soft tissue. A real zygoma is covered by soft tissue; direct observation of the fracture line is not easy. During surgery, undue tension from soft tissue traction makes reduction and drilling procedures difficult. These may increase surgical inaccuracy and unpredictability.

## Conclusion

The results of a model surgery show that combining a presurgical CAD/CAM method and a navigation system to generate FNRPs for assisting secondary zygoma reduction is accurate and practical. Compared with a simulation plan, unexpected deviations for the reduced zygomas in the skull models were limited. However, the influence of soft tissue was not considered. Further application in clinical use is necessary to prove the proposed method’s usefulness in real surgery.
